# Pakistan’s Progress on Universal Health Coverage: Lessons Learned in Priority Setting and Challenges Ahead in Reinforcing Primary Healthcare

**DOI:** 10.34172/ijhpm.2024.8450

**Published:** 2024-04-16

**Authors:** Ala Alwan, Dean T. Jamison, Sameen Siddiqi, Anna Vassall

**Affiliations:** ^1^DCP3 Country Translation Project, London School of Hygiene and Tropical Medicine, London, UK.; ^2^University of California, San Francisco, CA, USA.; ^3^Department of Community Health Sciences, Aga Khan University, Karachi, Pakistan.; ^4^Global Health Economics Centre, London School of Hygiene and Tropical Medicine, London, UK.

**Keywords:** Universal Health Coverage, Primary Healthcare, Pakistan, Disease Control Priorities, Essential Health Package, EPHS

## Abstract

Pakistan developed an essential package of health services at the primary healthcare (PHC) level as a key component of health reforms aiming to achieve universal health coverage (UHC). This supplement describes the methods and processes adopted for evidence-informed prioritization of services, policy decisions adopted, and the lessons learned in package design as well as in the transition to effective rollout. The papers conclude that evidence-informed deliberative processes can be effectively applied to design affordable packages of services that represent good value for money and address a major part of the disease burden. Transition to implementation requires a comprehensive assessment of health system gaps, strong engagement of the planning and financing sectors, serious involvement of key national stakeholders and the private health sector, capacity building, and institutionalization of technical and managerial skills. Pakistan’s experience highlights the need for updating the evidence and model packages of the Disease Control Priorities 3 (DCP3) initiative and reinforcing international collaboration to support technical guidance to countries in priority setting and UHC reforms.

## Introduction

 Universal health coverage (UHC) is an overarching goal for global health and national health strategies. UHC means that all people should have access to the full range of quality healthcare services they need, where and when they need them, without facing financial hardship.^1^ It covers the full continuum of health services, from health promotion to prevention, treatment, and rehabilitation. In 2015, all countries committed to achieving UHC as part of the Sustainable Development Goals (SDGs).^2^ Achieving UHC calls for proactive measures across its three key dimensions: extending population coverage, expanding the range of health services, and reducing financial risk.

 Eight years after their endorsement, the world is off track in making significant progress towards the SDG targets. Global commitment on UHC was reaffirmed in 2019 by Heads of State and Government, who pledged to scale up efforts on UHC in a special high-level meeting of the United Nations (UN) General Assembly.^3^ Yet, the 2023 Global Monitoring Report on Tracking UHC^4^ provides alarming signals. In 2021, around 4.5 billion people were not fully covered by essential health services and over one billion people experienced catastrophic health spending.^4^ Out of 194 UN member states, 108 have experienced no significant change or even a decline in service coverage since the SDG launch. Globally, the African and Eastern Mediterranean regions are facing the lowest service coverage and financial protection levels.

 To reinvigorate the global response, the UN convened a second high-level meeting on September 21, 2023. However, the resulting political declaration suggests continuing onwards as is, and unfortunately no new approaches to accelerate the pace of UHC were proposed within its over 60 actions recommended to reinforce efforts in countries.^5^

 Pakistan, the world’s fifth most populous country, is committed to achieve the UHC target in its National Health Vision.^6^ The Inter-Ministerial Health and Population Council endorsed, in 2018, a national initiative to assess the coverage of existing health interventions and set an evidence-informed, affordable, and feasibly implemented package of essential health services in order to achieve UHC through a primary healthcare (PHC) approach. The collection of five papers in this supplement presents the experience of Pakistan and reviews the decision-making processes, the challenges ahead, including the requirements for the transition from package design to effective rollout.^7–11^

 Pakistan faces considerable challenges in enhancing the health of its rapidly increasing population. While significant progress in improving key health indicators has been achieved, the reported UHC service coverage index^[1]^ remains low at 45.^12^ Over half of the total health expenditure is covered by out-of-pocket expenditure, with a significant proportion of the population experiencing catastrophic spending.

 Pakistan’s strategic approach on UHC reforms was to improve access to high-impact health services at the PHC level and to use an evidence-informed process to select essential services grounded on local needs and realities. The Inter-Ministerial Health and Population Council decided to use the evidence and model packages derived from the third edition of the Disease Control Priorities (DCP3)^13,14^ to guide country deliberations and decision-making processes. What makes DCP3 unique is the focus on UHC and on supporting low- and lower-middle income countries (LLMICs) in priority-setting and the development of essential health packages through a country translation approach.^15^ The concept offered by DCP3 is also consistent with Pakistan’s strategy on UHC and the focus on PHC; public financing of the highest-impact health interventions through domestic resources provides the most sustainable approach to implementing essential packages of health services (EPHS) for all.

 Pakistan developed its first evidence informed EPHS at the national and provincial levels in 2021 as the cornerstone of UHC reforms. The package design followed an inclusive and transparent process, led by the Federal Ministry of National Health Services, Regulations and Coordination, with engagement of a wide range of stakeholders and development partners.^7^ A systematic process was followed in designing the package, supported by sustained high-level political commitment and a governance structure for evidence-informed decision-making. The aim was to reach high-level consensus on affordable and feasibly implemented essential health services, accessible to all people as the central pathway to achieve UHC. The five papers in this collection discuss the timeline and steps involved in developing the Pakistan EPHS,^7^ the evidence-informed deliberative process, including the establishment of a governance structure and engagement of key national stakeholders,^8^ the methods applied in costing interventions,^9^ assessment of the interventions’ cost-effectiveness,^10^ and the prioritization decisions made throughout the different stages of package design.^11^ Box 1 presents the five papers outlining the experience of Pakistan.

**Box 1. List of Papers in This Collection**

1. Alwan A, Siddiqi S, Malik S, et al. Addressing the UHC challenge using the Disease Control Priorities 3 approach: experience from Pakistan and lessons learned. *Int J Health Policy Manag. * 2023;12:8003. doi:10.34172/ijhpm.2023.8003
2. Baltussen R, Jansen M, Akhtar SS, et al. The use of evidence-informed deliberative processes for designing the essential package of health services in Pakistan. *Int J Health Policy Manag. *2023;12:8004. doi:10.34172/ijhpm.2023.8004
3. Raza W, Zulfiqar W, Shah MM, et al. Costing Interventions for Developing an Essential Package of Health Services: Application of a Rapid Method and Results from Pakistan. *Int J Health Policy Manag.* 2024;13:8006. doi:10.34172/ijhpm.2023.8006
4. Huda M, Kitson N, Saadi N, et al. Assessing global evidence on cost-effectiveness to inform development of Pakistan’s essential package of health services. *Int J Health Policy Manag. *2024;13:8005. doi:10.34172/ijhpm.2023.8005
5. Torres-Rueda S, Vassall A, Zaidi R, et al. The use of evidence to design an essential package of health services in Pakistan: a review and analysis of prioritisation decisions at different stages of the appraisal process.* Int J Health Policy Manag. *2024;13:8043. doi:10.34172/ijhpm.2024.8043

## Lessons Learned and Key Challenges

 In this collection, Alwan et al^7^ provides an overview of the rationale, aims, and approach followed to construct a set of publicly-financed essential health services, affordable to Pakistan, that can be rolled out to reinforce PHC and accelerate progress toward UHC. The result of the national initiative was a package that received full endorsement by the government at the federal and provincial levels. Despite the successful outcome, the authors also review the lessons learned during the design process and the challenges encountered in the transition to package implementation.

 A critical challenge in many LLMICs designing packages is that they are not successfully implemented and are therefore not accessible to all segments of the population. This situation is particularly seen if high-level commitment is absent, when package design is weak and inappropriate, or if the recommended package is unaffordable and fails to consider the health system’s capacity for effective implementation.^7,16^

 Pakistan made special efforts to learn from the experience in other countries and to invest in a realistic and affordable package, yet there were missing areas in the design process that would have contributed more effectively to package implementation. Specifically, benefit package implementation requires a substantive and serious dialogue with community and civil society representatives, better availability of local epidemiological, service and cost evidence, a strong engagement of the planning and financing government sectors, a deep involvement of the private health sector and a more comprehensive health system assessment. The Pakistan experience highlighted the critical need to clearly identify and address the health system gaps hindering package rollout from the outset of package development.^7^

 The papers also provide additional lessons for Pakistan and other countries on the prioritization process. For example, Baltussen et al^8^ conclude that despite scarcity of local data, evidence-informed deliberative and decision-making processes can be effectively applied in a transparent and participatory manner in LLMICs to design essential services that are affordable and represent good value for money. Torres-Rueda et al^11^ examined the trends in the composition of the package during different phases of the priority setting process and found that the value for money for the interventions included generally increased throughout the process, illustrating that economic evidence can inform prioritization. However, interventions with high current coverage, regardless of cost-effectiveness, were overwhelmingly prioritized for inclusion, showing the importance of the current context. While this trade-off may suggest a possible aversion to disinvestment, issues around intervention feasibility were important considerations for policy-makers; and may result in a more impactful package.

 Raza et al^9^ detail the process and methodologies used in estimating the cost of the Pakistan EPHS and conclude that full transparency in methodological approaches and assumptions and systematically conducting sensitivity analyses are critical. Their work drew on but went beyond DCP3’s methods and data on cost.^17^ This conclusion is also supported by a recently published analysis of costing processes in five African and Eastern Mediterranean countries, including Pakistan.^18^ The analysis also shows substantial differences in the way methodologies were implemented across countries, particularly in relation to health system costs, highlighting that costs cannot be directly translated into budgets without further adjustments.

 A key decision criterion to prioritize health interventions is cost-effectiveness. Huda et al^10^ examine the usefulness of global evidence on cost-effectiveness in supporting decision-making in Pakistan, where only a small proportion of the prioritized interventions could be supported with evidence that was directly applicable to the country context. The paper provides transparency around the challenges associated with transferability of global evidence and highlights the need for further development of country-driven participatory approaches to be used alongside global resources such as DCP3, for localizing global and regional economic evaluation evidence.

## Implications for Updating the DCP3 Evidence and Model Packages

 The experience in Pakistan and other countries participating in the DCP3 country translation review initiative highlights the invaluable technical guidance provided by the DCP3 evidence in support of countries committed to reinforce action on UHC.^19^ However, the experience also holds important implications for updating the DCP3 evidence and model packages to better serve the needs and financial realities in LLMICs.

 First, the DCP3 evidence on cost and cost-effectiveness could only be partially attributed to regionally and locally generated studies^10^; this highlights the need for international and regional collaboration in building capacity in LLMICs to generate locally relevant evidence and/or methods for transferring global evidence. The forthcoming first volume of DCP4 reviews national efforts in constructing health benefit packages and the need for further investment. DCP4 aims to include an in-depth analysis of the areas where local evidence is needed and recommend options for reinforcing national capacities in LLMICs.

 Second, the DCP3 Essential UHC package, while a valuable tool in guiding prioritization of services in LLMICs, may benefit from a more specific definition of interventions. Particular attention should be paid to interventions that are too generic or have multiple components requiring several clinical actions, which can be challenging to assess in the same way as single disease focused interventions.

 Third, the model packages should be reviewed to include critical interventions which are currently missing, like emergency medical services and pandemic preparedness and response. Fourth, public sector health expenditures outside the package need spotlighting. These are, by definition, also a priority to countries and they claim substantial resources. Fifth, the experience in Pakistan and other countries underscores the fiscal constraints faced by LLMICs, which required governments to adopt rigorous prioritization processes to come up with realistically affordable packages of essential health services that are considerably less ambitious than the DCP3 Essential UHC and even the High Priority Package in terms of number of interventions and costs.

## Recommendations to LLMICs and Future Directions

 Developing and implementing publicly funded packages of essential health services serve as a linchpin for achieving equitable access to essential services while also reducing the financial burden associated with healthcare. To address the UHC dimensions, LLMICs must invest in evidence-informed, high-impact health services that take into account health system realities and the available fiscal space for health. Key to developing an affordable and viable package is to follow a sound package design process. Experience in Pakistan and other countries developing UHC packages^7,16^ shows that strong political commitment and competent national leadership is a must for sound and effective package design. Early engagement of key national stakeholders, especially of the Planning and Finance sectors, and the private health sector is of crucial importance. Assessments of health system performance and capacity and in-depth analyses of the available fiscal space for health and financing mechanisms are critical steps at the onset of package design. The strategic framework proposed by DCP3 to ensure country readiness for proper UHC package design and the evidence-informed deliberative process outlined in this collection offer guidance to LLMICs in prioritizing health services for public financing.^7,8,16^

 Most LLMICs engaged in reinforcing efforts to achieve UHC require critical technical and institutional capacities, which most of them currently lack. Too often, existing technical support is inadequate, fragmented, and fails to build local capacity or institutionalize basic skills. Countries need to be well equipped with the knowledge and good practice on how to assess their health systems, prioritize high-impact services, construct an affordable and feasibly implemented package of essential services and address health system gaps that hinder implementation. These are important realities that underscore the need for international collaboration engaging relevant academic institutions, health-oriented multilateral agencies, and development partners to establish a more effective model to reinforce technical guidance for LLMICs in priority setting, health financing, and UHC package design and implementation. A three-pronged strategy to reinforce technical cooperation with LLMICs has recently been proposed by the DCP3 Country Translation Project for discussion.^20^

## Ethical issues

 Not applicable.

## Competing interests

 Authors declare that they have no competing interests.

## Funding

 DCP3 Country Translation Project is funded by the Bill & Melinda Gates Foundation (Grant OPP1201812). The funder had no role in study design, data collection and analysis, decision to publish, or preparation of the manuscript.

## Endnotes

 UHC service coverage index (SDG 3.8.1) combines 14 tracer indicators of service coverage into a single summary measure. The index is reported on a unitless scale ranging from 0 to 100.

## References

[1] World Health Organization (WHO). Universal Health Coverage (UHC). https://www.who.int/news-room/fact-sheets/detail/universal-health-coverage-(uhc). Accessed October 20, 2023. Published 2023.

[2] United Nations. The UN Sustainable Development Goals. http://www.un.org/sustainabledevelopment/summit/. Accessed January 9, 2023. Published 2015.

[3] United Nations General Assembly. Political Declaration of the High-Level Meeting on Universal Health Coverage “Universal Health Coverage: Moving Together to Build a Healthier World.” https://www.un.org/pga/73/wp-content/uploads/sites/53/2019/07/FINAL-draft-UHC-Political-Declaration.pdf. Published 2019.

[4] World Health Organization (WHO), The World Bank. Tracking Universal Health Coverage: 2023 Global Monitoring Report. Geneva, Switzerland: WHO, The World Bank; 2023. https://www.who.int/publications/i/item/9789240080379.

[5] United Nations. Political Declaration of the High-Level Meeting on Universal Health Coverage “Universal Health Coverage: Expanding Our Ambition for Health and Well-Being in a Post-COVID World.” https://www.un.org/pga/77/wp-content/uploads/sites/105/2023/09/UHC-Final-Text.pdf. Published 2023.

[6] Government of Pakistan. Pakistan National Health Vision (2016-25). https://extranet.who.int/countryplanningcycles/sites/default/files/planning_cycle_repository/pakistan/national_health_vision_2016-25_30-08-2016.pdf. Accessed October 20, 2023. Published 2016.

[7] Alwan A, Siddiqi S, Safi M (2023). Addressing the UHC challenge using the Disease Control Priorities 3 approach: lessons learned and an overview of the Pakistan experience. Int J Health Policy Manag.

[8] Baltussen R, Jansen M, Akhtar SS (2023). The use of evidence-informed deliberative processes for designing the essential package of health services in Pakistan. Int J Health Policy Manag.

[9] Raza W, Zulfiqar W, Shah MM (2024). Costing interventions for developing an Essential Package of Health Services: application of a rapid method and results from Pakistan. Int J Health Policy Manag.

[10] Huda M, Kitson N, Saadi N (2024). Assessing global evidence on cost-effectiveness to inform development of Pakistan’s Essential Package of Health Services. Int J Health Policy Manag.

[11] Torres-Rueda S, Vassall A, Zaidi R (2024). The use of evidence to design an essential package of health services in Pakistan: a review and analysis of prioritisation decisions at different stages of the appraisal process. Int J Health Policy Manag.

[12] World Health Organization (WHO). UHC Service Coverage Index (SDG 3.8.1). https://www.who.int/data/gho/data/indicators/indicator-details/GHO/uhc-index-of-service-coverage. Accessed September 26, 2023. Published 2021.

[13] Jamison DT, Gelband H, Horton S, et al. Disease Control Priorities: Improving Health and Reducing Poverty. Vol 9. 3rd ed. Washington, DC: World Bank; 2018. 10.1596/978-1-4648-0527-1. 30212058

[14] Jamison DT, Alwan A, Mock CN (2018). Universal health coverage and intersectoral action for health: key messages from Disease Control Priorities, 3rd edition. Lancet.

[15] Disease Control Priorities 3 (DCP3). DCP3 Country Translation Project. http://dcp-3.org/translation. Accessed August 20, 2023.

[16] Alwan A, Majdzadeh R, Yamey G (2023). Country readiness and prerequisites for successful design and transition to implementation of essential packages of health services: experience from six countries. BMJ Glob Health.

[17] Watkins DA, Qi J, Kawakatsu Y, Pickersgill SJ, Horton SE, Jamison DT (2020). Resource requirements for essential universal health coverage: a modelling study based on findings from Disease Control Priorities, 3rd edition. Lancet Glob Health.

[18] Gaudin S, Raza W, Skordis J, Soucat A, Stenberg K, Alwan A (2023). Using costing to facilitate policy making towards universal health coverage: findings and recommendations from country-level experiences. BMJ Glob Health.

[19] Alwan A, Yamey G, Soucat A (2023). Essential packages of health services in low-income and lower-middle-income countries: what have we learnt?. BMJ Glob Health.

[20] Disease Control Priorities 3 (DCP3). Reinforcing Priority Setting and Design and Delivery of Health Services Packages for Universal Health Coverage: A Strategy for a New and Sustainable Technical Cooperation Model in Low- and Lower Middle-Income Countries. Geneva, Switzerland: DCP 3; 2023. https://www.dcp-3.org/resources/strategy-priority-LLMICs.

